# Evaluating pregnancy termination decisions for fetal anomalies: a retrospective study in a tertiary referral center

**DOI:** 10.1590/1806-9282.20231118

**Published:** 2024-05-20

**Authors:** Gokhan Bolluk, Isil Turan Bakirci, Mehmet Cok, Handan Turhan Karakus, Kani Sengonul

**Affiliations:** 1 Basaksehir Cam and Sakura City Hospital, Department of Obstetrics and Gynecology, Division of Perinatology – İstanbul, Turkey.; 2 Basaksehir Cam and Sakura City Hospital, Department of Obstetrics and Gynecology – İstanbul, Turkey.; 3 Sorgun State Hospital, Department of Obstetrics and Gynecology – Yozgat, Turkey.

**Keywords:** Congenital anomalies, Induced abortion, Maternal health, Fetus, Prenatal diagnoses

## Abstract

**OBJECTIVE:**

The aim of this study was to examine the factors that influence pregnancy termination due to fetal anomalies, regardless of gestational age, within the legal framework of Turkey.

**METHODS:**

This retrospective study was conducted between January 2021 and July 2023 at a tertiary perinatology center to analyze patients undergoing pregnancy termination. The process involved multidisciplinary evaluations and informed consent, resulting in 326 pregnancy terminations, categorized by gestational timing.

**RESULTS:**

Of the 326 patients studied, 219 opted for terminations. Gestational week at diagnosis significantly influenced the decision to terminate, with fetal anomalies being the primary indication. Chromosomal abnormalities accounted for 15.9% of the cases, while structural anomalies and maternal disorders accounted for 84.1% and structural malformations accounted for 84.1% of the cases. Late terminations (≥23 weeks) accounted for 30% of cases and required complex procedures.

**CONCLUSION:**

The findings of this study indicate that maternal demographic factors have a limited impact on termination decisions. Early diagnosis of fetal anomalies is crucial for informed decision-making and emotional support, and the psychological consequences of late termination highlight the need for maternal support. Obstetricians play a vital role in facilitating early intervention. This study underscores the complex medical, ethical, and psychological aspects of pregnancy termination due to fetal anomalies. It emphasizes the importance of a holistic approach, considering medical, ethical, and psychological factors and the crucial role of healthcare professionals in supporting families during this challenging process.

## INTRODUCTION

In recent years, significant transformations have occurred in the medical field with the widespread adoption of ultrasound technology in gynecology and obstetrics and advancements in diagnostic devices. Advances in laboratory technologies, particularly in the early stages of pregnancy, have facilitated the early detection of fetal structural and genetic anomalies. In conjunction with establishing universal prenatal screening policies, these advancements have enabled timely management decisions at earlier gestational weeks. When confronted with severe or fatal abnormalities devoid of viable intrauterine treatments, families are often offered the option of terminating pregnancy. Numerous factors, including gestational age, anomaly severity, systemic involvement, and chromosomal abnormalities, influence decisions regarding termination^1^.

Within the framework of official laws and specific conditions defined in our country, the choice of pregnancy termination in response to fetal anomalies is extended to parents. The legal gestational age for permissible termination varies across countries. While some countries do not impose a gestational week restriction for pregnancy termination because of fetal anomalies, others prohibit this practice^2-5^.

In our country, the "Family Planning Law," enacted on May 24, 1983, allows elective termination until the 10th week of gestation^2^. After this period, termination can be performed without gestational week limitations if the pregnancy risks the woman’s health and has a high likelihood of severe disability or an incurable fatal disease in the fetus. Notably, there was no upper gestational age limit for pregnancy termination. When maternal or fetal grounds warrant termination, the decision requires concurrence from a panel of at least three specialists.

The primary objective of this study was to comprehensively investigate and assess pregnancies in our clinic, which provided the opportunity for pregnancy termination due to fetal anomalies, regardless of the gestational week.

## METHODS

This retrospective study was conducted in the Department of Obstetrics and Gynecology of a leading tertiary referral center for perinatology. Patients who underwent pregnancy termination for fetal anomalies and maternal medical conditions between January 2021 and July 2023 were analyzed. The study strictly adhered to the ethical principles of the Declaration of Helsinki.

Perinatologists used the Arietta 850 USG system (Hitachi, Japan) for fetal ultrasonographic evaluations and preliminary diagnoses. In cases where fetal anomalies were confirmed, families were reevaluated by a multidisciplinary committee of experts in medical genetics, pediatric surgery, pediatric nephrology, pediatric cardiology, pediatric neurology, and neurosurgery. These evaluations were conducted within the genetics or cardiology council of the perinatology clinic, where families are informed of their diagnoses and prognoses.

Following comprehensive deliberation and obtaining informed consent from the cases reviewed by the committee, 326 cases were presented with the option of pregnancy termination, regardless of gestational week. Pregnancy termination plans were subsequently developed for the patients who consented to the procedure.

The cases were classified based on gestational timing, with pregnancies exceeding 23 weeks as "late termination" and those below 23 weeks as "early termination." Detailed discussions occurred with expectant mothers concerning pregnancy termination methods and the associated risks. The preferred termination method at our hospital involves medical termination using vaginal misoprostol (Cytotec; 200 μg tablet, Ali Arif, Istanbul), with or without oxytocin induction. In cases of "late termination" (≥23 weeks), fetal demise was ensured through intracardiac administration of potassium chloride under ultrasound guidance.

The International Classification of Diseases: Version 10 (ICD-10) was used to categorize the termination indications in this study. Hospital database records were used to retrieve each case’s medical and demographic data. Prenatal karyotype analysis was recommended for all terminations except in instances dictated by maternal indications. After the termination of pregnancy, a fetal autopsy was performed in cases that accepted the autopsy, and the families were given comprehensive information about the procedures to be performed. Termination of pregnancy was initiated after obtaining signed consent from all parents.

The study was conducted with the approval of the Ethics Committee of Basaksehir Cam and Sakura City Hospital (KAEK/27.09.2023.433).

### Statistical analysis

Statistical analysis was performed using the chi-squared test or Fisher’s exact test for categorical variables, and univariate analysis was conducted using the Student’s t-test or Mann–Whitney U test for factors that may correlate with the outcome being terminated. Descriptive statistics are presented as percentages, means±standard deviations, and medians (min–max). The results were analyzed using IBM SPSS Statistics for Windows, Version 26.0, and statistical significance was set at p<0.05.

## RESULTS

Table 1 summarizes the demographic characteristics of the 326 patients offered the option to terminate the pregnancy. Of these patients, 107 chose not to terminate the pregnancy, whereas 219 opted for termination and underwent the procedure.

**Table 1:** The demographic characteristics of 326 patients offered the option to terminate the pregnancy.

Characteristics	Mean±SD	Median (min–max)
Age	28.6±7.5	28 (1–46)
Gravidity	2.4±1.7	2 (0–13)
Parity	1.0±1.2	1 (0–9)
Miscarriage	0.5±0.9	0 (0–5)
Number of children	1.0±1.2	1 (0–9)
GA at diagnosis (week)	21.6±6.3	21 (2–37)
GA at TOP (week)	20.0±4.7	20.5 (11–33)

GA: gestational age; TOP: termination of pregnancy; SD: standard deviation.

Table 2 compares the characteristics of the declined termination (n=107) and termination acceptance groups (n=219).

**Table 2:** Comparison between cases that declined and those that accepted pregnancy termination.

	Declined termination (n=107)		Accepted termination (n=219)		p-value
Age	28.23±7.35	28 (2–46)	28.84±7.56	28.5 (1–45)	0.490
Gravidity	2.25±1.47	2 (0–7)	2.54±1.84	2 (0–13)	0.302
Parity	0.9±1.14	0 (0–5)	1.04±1.24	1 (0–9)	0.271
Miscarriage	0.37±0.77	0 (0–4)	0.5±0.92	0 (0–5)	0.292
Number of children	0.87±1.14	0 (0–5)	1.03±1.24	1 (0–9)	0.183
GA at diagnosis (week)	26±6.71	27 (2–37)	19.43±4.77	20 (10–33)	**<0.001**
GA at TOP (week)	–	–	19.97±4.74	20.5 (11–33)	NA

GA: gestational age; TOP: termination of pregnancy. NA indicates data not applicable or not available. Data are presented as the mean with standard deviation and the median with minimum and maximum. Significances are presented in bold.

In total, 219 pregnancies were terminated, including 215 singleton pregnancies and four twin pregnancies. The mean age of the 219 patients who underwent termination was 28.84±7.56. The average gestational week at diagnosis was 19.43±4.77, and the mean gestational week at the time of termination was 19.97±4.74 for those who underwent termination. In contrast, the mean gestational age at diagnosis in the non-termination group was 26±6.71. Notably, only the gestational week for termination showed a significant difference between the two groups. The median gestational age in the non-terminated group was significantly higher than that in the terminated group (p<0.001) (see Table 2).

Of the 219 pregnancy terminations, 70% (153 cases) were categorized as early terminations (<23 weeks) because of fetal anomalies or maternal indications. Karyotype analysis was conducted in 21.9% (48 cases), revealing chromosomal or genetic anomalies in 35 cases. Trisomy 21 was the most common chromosomal anomaly, followed by trisomy 18 and 13. Distinct genetic disorders were also identified, including nail patella syndrome, Meckel–Gruber syndrome, and deletions in smn1. Postmortem autopsy was performed in 2.2% (5 cases) of the cases, confirming concordance with the prenatal findings.

Termination indications primarily comprised structural anomalies and maternal disorders, accounting for 84.1% of cases. Central nervous system (CNS) anomalies, primarily neural tube defects, constituted the majority (50.6%) of terminations. Chromosomal abnormalities were the second most prevalent cause (16%), followed by genitourinary system anomalies (10%). The isolated abdominal wall or gastrointestinal anomalies did not lead to termination.

The gestational weeks ranged from the 11th week due to maternal interstitial lung disease to the 33rd week in patients with trisomy 21. The misoprostol protocol was primarily used for terminations before the 23rd gestational week, whereas those beyond the 23rd week underwent a feticide procedure followed by the misoprostol protocol. However, exceptions were noted in five cases that required hysterotomy. No uterine ruptures or hysterectomies were performed.

The study included four twin pregnancies: one terminated due to conjoined twins (thoracopagus) and the others due to dichorionic diamniotic twins. Structural anomalies prompted the termination of a single fetus in these cases. The distribution of terminations based on the specific indications is presented in Table 3.

**Table 3:** Distribution of pregnancy terminations according to fetal and maternal indications.

		Number of cases	Percentage of total (%)
Central nervous system		111	50.7
	Neural tube defects	49	22.4
	Anencephaly	31	14.2
	Hydrocephaly	7	3.2
	Encephalocele	11	5.0
	Agenesis of corpus callosum	9	4.1
	Other	4	1.8
Multiple anomalies		10	4.6
Hydrops fetalis		7	3.2
	Limb body wall complex	3	1.4
Chromosomal anomalies–genetic diseases		35	16.0
Trisomy 21		22	10.0
	Trisomy 18	7	3.2
	Trisomy 13	3	1.4
	Other	3	1.4
Genitourinary system anomalies		21	9.6
Renal agenesis		11	5.0
	Multicystic dysplastic kidney	10	4.6
Skeletal system		8	3.6
Cardiovascular system anomalies		5	2.2
Hypoplastic left heart syndrome		3	1.37
Pulmonary atresia–hypoplastic right ventricle		2	0.91
Head and neck anomalies		4	1.8
Maternal disorders		6	2.8
Breast cancer		4	1.8
	Maternal severe heart disease	1	0.5
	Maternal lung disease	1	0.5
Other (anhydramnios, conjoined twin)		19	8.7
Total		219	100

## DISCUSSION

This study presents a comprehensive review of pregnancy termination indications and methods by trimester and offers valuable insights. Demographic analysis revealed no significant differences between the termination and continuation groups regarding age, pregnancy, or living children. Maternal age, reproductive history, and pregnancy count minimally affected the termination decisions.

This study aimed to explore variables influencing pregnancy termination decisions. Among the 326 participants, 219 terminated pregnancies, while 107 continued pregnancies. Those declining terminations had higher gestational age, emphasizing their role in decision-making; the likelihood of termination decreased as gestational age increased.

Our study confirmed that fetal structural malformations were the primary reason for termination^6^. CNS and genitourinary anomalies were the predominant anomalies. Chromosomal/genetic factors constituted 16% of the reasons for termination.

Karyotype analysis, performed on 35 patients, identified 16% chromosomal/genetic anomalies, including trisomy 21, 18, and 13, and genetic disorders. Karyotyping aids families in informed decision-making. The fetal autopsy rate in our study was 2.5%, which is lower than the rates reported in the literature^7,8^. This decrease was attributed to healthcare professionals’ and families’ misunderstandings of the procedure, cultural and religious beliefs, emotional difficulties, and accessibility issues. The emergence of new diagnostic methods may further reduce the need for autopsy. Therefore, it is essential to address this issue by increasing awareness, education, and communication.

Early detection of fetal anomalies is crucial for making informed decisions regarding pregnancy termination, optimizing diagnosis and medical procedures, and offering support to families. In our study, we found that 70% of early terminations (<23 weeks) and 30% of late terminations (≥23 weeks) were performed because of severe fetal anomalies or maternal indications requiring feticide.

Turkey has witnessed a decline in late pregnancy terminations from 46.2 to 30% over the past 16 years, primarily owing to improved screening and healthcare services^9^. However, the 30% late termination rate raises concerns about the effectiveness of 11–14-week examinations.

Termination of pregnancy has been noted to have profound psychological effects, with research showing that individuals who undergo this procedure are more likely to show symptoms of post-traumatic stress disorder (PTSD) and experience depression, especially when the gestational age is advanced^10-15^. Healthcare professionals must prioritize understanding the psychological dimensions of this process, offering support, and promoting maternal mental health. Although medical advances may reduce the need for termination, early anomaly identification remains crucial. Genetic screening may shift the indications for termination from structural to genetic issues and from late to early termination. Legal flexibility at an advanced gestational age is essential.

However, this study has limitations, including its retrospective design, single-center focus, and potential cultural and ethical influences.

## CONCLUSION

This study provides valuable insights into pregnancy termination, emphasizing the need for a holistic approach that considers medical, ethical, and psychological aspects and the critical role of early diagnosis. An immediate focus should be on timely anomaly management.
